# Two-Dimensional Thin Layer Chromatography-Bioautography Designed to Separate and Locate Metabolites with Antioxidant Activity Contained on* Spirulina platensis*

**DOI:** 10.1155/2018/4605373

**Published:** 2018-07-05

**Authors:** Margarita Cid-Hernández, Fernando Antonio López Dellamary-Toral, Luis Javier González-Ortiz, María Judith Sánchez-Peña, Fermín Paul Pacheco-Moisés

**Affiliations:** ^1^Departamento de Química, Centro Universitario de Ciencias Exactas e Ingenierías, Universidad de Guadalajara, Blvd. Marcelino García Barragán 1421, 44430 Guadalajara, Jalisco, Mexico; ^2^Departamento de Madera, Celulosa y Papel, Universidad de Guadalajara, km 15.5 de la Carretera Guadalajara-Nogales, 45220 Zapopan, Jalisco, Mexico

## Abstract

*Spirulina platensis *contains several biologically active compounds, some of them with antioxidant activity. Nevertheless, not all of these compounds have been identified to date. As a first step to achieving such identification, a methodology to perform two-dimensional thin layer chromatography bioautographies on silica gel thin layer chromatography plates was proposed. Starting with a reference binary system, 5 other binary systems were tested, in which the relative polarity was systematically increased. To further improve the separation behavior, a phase modifier (NH_4_OH) was used. The best separation results were obtained with the isopropyl alcohol/ethyl acetate/NH_4_OH ternary system. This experimental system allowed four well-resolved spots showing antioxidant activity as well as two additional areas with mixtures containing antioxidant compounds. Although the proposed methodology was designed with a specific application, it would be predictable that its field of use could be considerably greater, making the convenient modifications on the solvent polarity and “masking level” produced by the ammonium derivatives.

## 1. Introduction

*Spirulina platensis* is a cyanobacterium that has been used in Mexico and other countries since ancient times [1, 2]. Recently, it has attracted worldwide attention due to its potential as a protein source [2, 3] and its therapeutic properties (e.g., immunomodulatory functions) [4].* Spirulina *contains several known antioxidants, e.g., chlorophyll a, carotenoids, phycocyanins, glutathione, tocopherol, and others [5–7]. The relative amount of these compounds, as well as the possible presence of some additional ones, mainly depends on the environmental conditions during its culture [8–11]. The antioxidant activity of* Spirulina platensis* or its extracts has been reported for* in vitro* [9–12] and* in vivo *[13, 14] models. However, it is clear that such activity has been produced by relatively complex mixtures that contain an unidentified number of components, most of which could possess low or negligible antioxidant activity. Due to the high number of components potentially present in* Spirulina* samples, it would be predictable that, to take advantage of beneficial properties of* Spirulina*, the isolation and characterization of its components as much as possible will be necessary.

Thin layer chromatography (TLC) is known as a very useful technique, utilized to separate complex mixtures, whose behavior depends on the balance of hydrophobic, hydrophilic, and steric interactions, as well as hydrogen bonding, occurring between the analytes and the mobile and stationary phases [15]. The most used stationary phase is the silica gel [16–18], which contains Si atoms bonded to none, one, or two hydroxyl groups [19]. Silanol groups (Si-OH) present on silica can be reversibly dehydrated, producing a siloxane group (Si-O-Si) from two Si-OH groups [16]. Therefore, silica surface must simultaneously contain both groups, being their relative amount dependent on the amount of the cationic groups which came from the phase modifier (e.g., ammonium hydroxide or salts), which are interacting with the silanol groups, decreasing the retention trend of the stationary phase toward the metabolites that are being separated by the TLC technique [20]. Since only the Si-OH groups are considered as strong adsorption sites [17, 21], the retention behavior can be externally modified (e.g., when water molecules are absorbed, preferentially on the Si-OH groups) [22]. Thus, depending on the stationary phase characteristics, as well as the mobile phase and sample compositions, the phase modifiers (e.g., ammonium salts or derivatives) may interact with the Si-OH groups and, therefore, modify the analytes retention behavior, especially with polar and basic compounds [18, 23].

In certain complex systems (e.g., those containing components covering a wide spectra of polarities), to improve the separation, it could be useful to simultaneously use two different chromatographic systems (e.g., different pairs of stationary phase/mobile phase). That is, a normal stationary phase (silica gel) developed with a nonaqueous mobile phase, which separates preferably nonpolar components and, in parallel, a reverse stationary phase (octadecyl silica) developed with an aqueous phase, used to separate preferably polar components [24, 25]. However, it is clear that this methodology duplicates the human and material cost of the process, which is an important factor to be considered when the objective is to obtain a considerable mass of the isolate components (e.g., to analyze them). An alternative methodology requires the use of a dual phase TLC plate, which have allowed complex mixtures to be separated, where great separation selectivity was reached [24, 25]. Dual phase TLC plates contain a narrow zone of SiO_2_ and a wide zone of octadecyl silica (or vice versa). Unfortunately, at least in North America, these multi-K dual phase TLC plates are no longer available. Therefore, an alternative approach to effectively separate the compounds present in complex mixtures is to modify the interactions of the stationary phase, by means of the addition of a modifier to the mobile phase. Some amines have been used as phase modifiers, since they are able to mask silanol groups, reduce the silanophilic interactions with analytes, and, therefore, increase their retention factors (R_F_) [20, 26, 27]. Furthermore, it has been reported that the chromatographic resolution is strongly affected by the pK_a_ of the amine used. In fact, the use of basic systems promotes the separation of closely related compounds with minor structural differences [28].

On the other hand, since component(s) that are practically incompatible(s) with the mobile phase remain very close to the bottom of the thin layer chromatograms, the presence of spots containing two or more compounds at R_F_ values near to zero could be possible. An equivalent statement can be expressed about components that are very compatible with the mobile phase, which migrate up to the top of the chromatogram (R_F_=1.0). Therefore, to avoid the multicomponent spots presence, it is commonly preferred to consider it as well resolved, only those spots clearly separated (e.g., more distributed spots through the plate) that present retention factors not very near to zero or the unit [25, 29].

TLC bioautography (TLC bio) is an effective and inexpensive technique that combines the chromatographic separation with* in situ* localization of compounds with biological activity [30–32]. The reaction between the 1,1-diphenyl-2-picryl-hydrazyl (DPPH) radical and an active compound is a method commonly used to determine its antioxidant activity, which has been used to directly locate those types of compounds on TLC plates [33–37]. The characteristic reaction of this technique produces a pale yellow on the spots that contain compounds with antioxidant activity [34, 38].

Zarzycki* et al. *[33, 39] implemented a one-dimensional TLC technique (1D-TLC) to separate the chemical components present in four pharmaceutical formulations of* Spirulina platensis*. The cyanobacterium samples were extracted with methanol, acetone, or tetrahydrofuran, obtaining the best results with methanol [39, 40]. For all extracts, the spots on TLC plates were initially visualized using natural light and, to visualize additional spots, TLC plates were exposed to iodine vapors. Unfortunately, iodine vapors can react with some metabolites, interfering with their antioxidant activity, therefore impeding their evaluation with the DPPH technique.

The objective of this study was to provide a simple and cheap 2D-TLC biomethod for separation of compounds with antioxidant activity contained in* Spirulina platensis*. The ease of sample preparation, the quickness of this method, and the repeatability of the retention factors are the major novelties of this work.

## 2. Experimental

### 2.1. Materials and Reagents

Methanol (MeOH; Golden Bell, analytical grade), acetone (AcO; Karal, purity: 90%), isopropyl alcohol (IOH; Fermont, purity: 99.9%), ethyl acetate (EA; Karal, purity: 99.5%), and n-hexane (n-Hx; Fisher Chemical, purity: 99.9%) were distilled prior to use. Ammonium hydroxide aqueous solution (Fermont, concentration: 25-30%) and 1,1-diphenyl-2-picrylhydrazyl (DPPH; Sigma-Aldrich, purity: 97%) were used as received. TLC aluminum sheets were 20 cm × 20 cm (Merck; 1 mm thick, silica gel 60 F254), which were heated during 30 min at 100°C in an oven (Felisa, Model 292) and maintained on a glass desiccator until their use.* Spirulina platensis* used for the experiments was purchased from Natura Vitalis® GmbH in the form of tablets (Original spiruletten-1700 tablets; 400 mg of* Spirulina platensis*/tablet).

### 2.2. Extract

25 g of* S. platensis* tablets was crushed to fine powder using mortar and pestle and transferred into an Erlenmeyer flask containing 500 mL of MeOH. This mixture was allowed to macerate for 48 hours under constant stirring. During maceration, the sample was protected from light and kept under a nitrogen atmosphere. The crude extract was filtered and concentrated with a rotary evaporator (Buchi R-3) to reduce the final volume to 125 mL.

### 2.3. TLC Chromatography

TLC strips (2 cm × 10 cm) and TLC plates (20 cm × 20 cm) were used for 1D- and 2D-TLC, respectively. Nine different solvent systems (Table 1) were tested as mobile phases (MPs); the MP-1 was reported previously by Zarzycki et al., which was considered as the starting mobile phase [39, 40].

For 1D-TLC, a cylindrical glass chamber (10 cm x 11 cm; D x H) was used; its temperature was kept at 30± 1°C. Ten *μ*L of the methanolic extract was spotted near the bottom of the TLC strip. Then, the solvent of the applied extract was evaporated completely at room conditions. 15 min before the TLC strip was developed, ten mL of the correspondent MP was poured inside the chamber. Immediately, the developed strips were dried at room temperature and were photographed under visible light (VL) or ultraviolet light at 366 nm (UVL_366_). A Chromato-Vue CC-20 ultraviolet chromatography viewer, equipped with a UV filter from Ultra-Violet Products Inc., was used to allow the direct observation of the irradiated strips.

For 2D-TLCs, 100 *μ*L of the methanolic extract was spotted near the bottom of the TLC plate; the solvent of the applied extract was evaporated completely at room temperature. A standard TLC glass chamber (rectangular TLC developing tank complete from Aldrich; 27 cm x 26.5 cm x 7.0 cm; L x H x W) was placed inside a recirculating water bath and kept at 30 ± 1°C; the temperature inside the TLC chamber was monitored continuously to avoid thermal variations. 100 mL of the corresponding mobile phase was added to this chamber. At the end of the first development, the plate was dried at room temperature and again placed in the chamber in a perpendicular direction from the original, to be developed in a second dimension. To finish the process, the plate was dried at room temperature and the plates were photographed under VL and UVL_366_ as above.

To corroborate the repeatability of the chromatographic procedure, the 2D-TLCs were performed by quintuplicate obtaining repetitive results, evaluated by their respective R_F_ values.

### 2.4. 2D-TLC Bioautographic Assay

In order to locate spots with probable antioxidant activity, the 2D-TLC plates were carefully dipped for 3-5 seconds in a methanolic solution of DPPH (0.25 mM) and dried at room temperature for 30 seconds. Then, the first photographic record was taken (t_0_); subsequently, more photographs were taken each hour, for 12 hours. Additional pictures were taken every 12 hours up to 48 hours and, for the MP-5c/MP-4a system (1^st^ development/2^nd^ development), some extra photographs were collected at longer times. Nevertheless, only the images taken at t_0_ and when the spots showed maximum intensity (t_F_; for the MP-1/MP-1 system, t_F_ =6 h and, for the MP-5c/MP-4a one, t_F_ =7 days) are shown.

## 3. Results and Discussion

### 3.1. 1D-TLCs

In order to separate the compounds of the methanolic extract of* Spirulina platensis*, a “family” of 1D-TLCs was prepared. For this purpose, just as starting point, the mobile phase used by Zarzycki et al. [39] was used, performing a systematic modification of such mobile phase to increase its relative polarity. The relative polarity of mobile phases was qualitatively estimated considering the solubility parameters of the correspondent pure solvents (*δ*); such criteria are commonly used to design binary solvents with a gradual decreasing of its solvation capacity [41]. This methodology considers that when the relative amount of the solvents is kept constant, using a solvent with a higher solubility parameter instead of another with a lower *δ* value produces a binary solvent with a higher polarity. Similarly, the polarity of the binary system increases as the relative content of the solvent with higher *δ* value increases [41].

**Figure 1:**
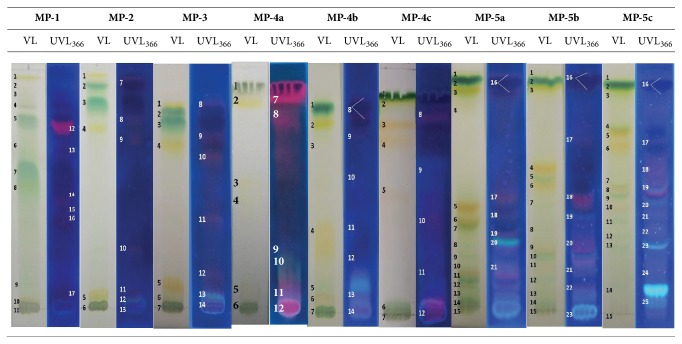
Representative photographs of 1D thin layer chromatograms for a methanolic extract of* Spirulina platensis*, developed with the indicated mobile phases and visualized under visible light (VL) or under ultraviolet light (UVL_366_).

Representative photographs of the TLC strips developed with the indicated mobile phases are shown in Figure 1. Under visible light, eleven spots were obtained when the MP-1 was used; this result was equivalent to the reported previously [39], demonstrating that such experiment was successfully reproduced. Besides, six additional spots were observed under UVL_366_. In contrast, only five additional spots were reported by Zarzycki when observed after iodine vapor exposure [39]. Nevertheless, since the well-resolved spots are especially important to this work, to establish the number of this type of spots is relevant. Thus, when analyzing the chromatograms obtained with the MP-1 (Figure 1) and their respective retention factors (Table 2), it was observed that only three well-resolved spots were obtained (since for spot # 1, R_F_= 0.97, there is uncertainty about whether that spot is a pure component or a mixture [28, 29]; therefore, this spot was not considered well resolved). When the behavior shown by the other binary mobile phases is considered (Figure 1 and Table 2), it can be observed that a considerably higher number of well-resolved spots was obtained, the best results being with the MP-4c, where 7 well-resolved spots can be observed. Thus, although the highest number of total spots was obtained with the MP-1, the highest number of well-resolved spots (the goal of this work) was obtained with the MP-4c, followed by the mobile phases MP-4a and MP-4b.

Besides, to make a pseudo-reverse phase that allows compounds with a high polarity to be resolved more adequately, a small amount of phase modifier (NH_4_OH) was added to the last three systems (MP-4a, b, and c), which promoted a considerable improvement in the chromatographic separation behavior (Figure 1). Thus, the number of total spots (T), as well as the number of well-resolved spots (W) obtained with phases MP-5a (T=21 and W=13), MP-5b (T=23 and W=11), and MP-5c (T=25 and W=18), was noticeably higher than the ones obtained with the equivalent phases without phase modifier (MP4a: T= 12 and W=5; MP-4b: T=14 and W=5; MP-4c: T=12 and W=7), which demonstrate the utility of the NH_4_OH presence in the system (Table 2). A global analysis of the 1D-TLC results shows that, with the MP-5c, a suitable balance of the interactions among the solvent system, the stationary phase (partially masked by the NH_4_OH), and the different metabolites was obtained, allowing for their gradual separation.

### 3.2. 2D-TLCs

**Figure 2:**
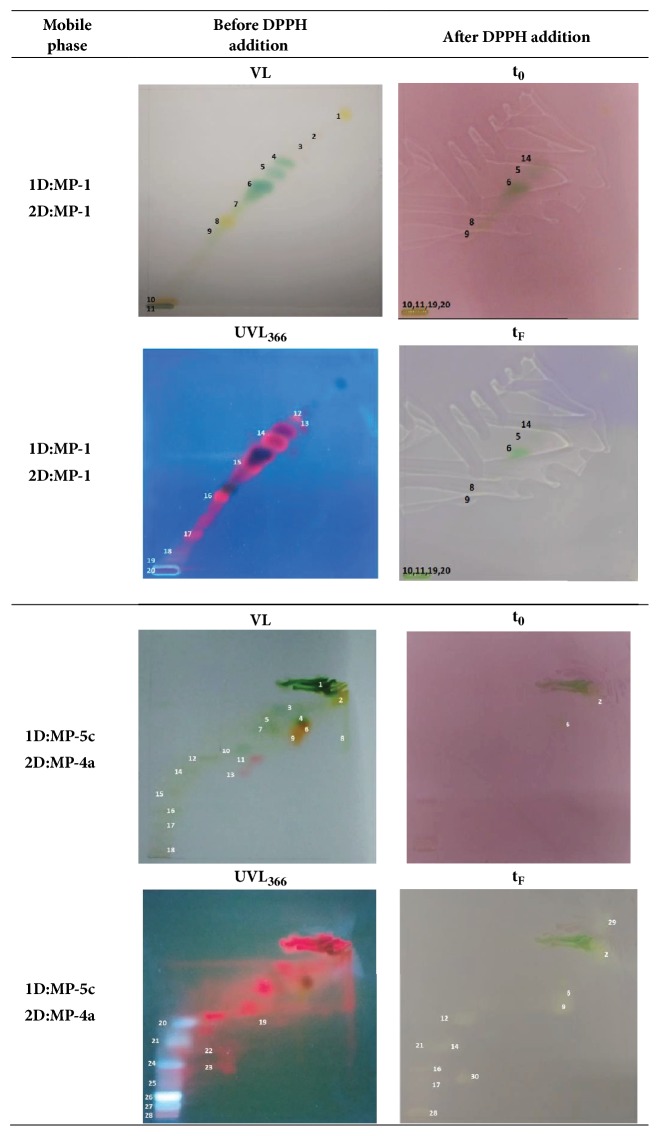
Representative photographs of 2D thin layer chromatograms for a methanolic extract of* Spirulina platensis*, developed with the indicated mobile phases and visualized under visible light (VL) and under ultraviolet light (UVL_366_) (central column) and the correspondent 2D thin layer biochromatograms, obtained after DPPH treatment at t_0_ and t_F_ (right column).

To further improve the resolution of the components contained in the methanolic extract of* Spirulina platensis*, a wide experimental set of 2D-TLCs was performed, obtaining the best results with the system that used in the first development (1^st^) the mobile phase named MP-5c and, in the second one (2^nd^), the MP-4a. Thus, in Figure 2 representative photographs of chromatographic plates developed by 2D-TLC are presented, which used the following solvent systems: (a) 1^st^: MP-1 and 2^nd^: MP-1 (herein referred to as the starting system) and (b) 1^st^: MP-5c and 2^nd^: MP-4a (herein referred to as the “best tested system”). Further, in Table 3 the corresponding R_F_ values for such experimental systems are presented.

Regarding the separating behavior of the starting system (a system of 2D-TLC using the same mobile phase in both developments), as it could be expected [42, 43], the separation quality was improved by the application of the second development. Thus, in the 1D-TLC that used the MP-1, seventeen spots were obtained, but only three well-resolved spots (Figure 1), whereas in the 2D-TLC, 20 total spots could be assessed and ten of them were considered well resolved (Figure 2).

In addition, a comparative analysis of the separating behavior of the last mentioned systems (Figure 2 and Table 3) shows that since the “best tested system” (MP-5c/MP-4a) exhibits 28 spots, with fifteen of them being considered well-resolved spots, it can be affirmed that its separation behavior represents a considerable improvement on the chromatographic resolution, when it is compared to the starting system (MP-1/MP-1; 20 total spots and 10 well-resolved ones).

### 3.3. 2D-TLC Bioautographic Assays

Since the main goal of this work is to identify well-resolved spots with antioxidant activity, in a preliminary step, the yellowish characteristic produced by the DPPH technique was looked for only on the well-resolved spots (Table 3). Thus, in Figure 2 it is observed that only spots # 6 and # 8 are useful for providing material susceptible to being used in a subsequent identification procedure (e.g., well-resolved and containing compounds with antioxidant activity). In an equivalent analysis, but with the MP-5c/MP-4a system, a higher number of useful spots were identified, specifically, spots #12, #14, #16, and #29. It is important to mention that spot #29 could be observed neither with visible light nor with UV light, but it could be observed when its component(s) reacted as a consequence of the DPPH addition. Taking into account only the above-mentioned information, the improvement reached with the proposed 2D-TLC bioautographic assays is evident.

A more in-depth analysis of the MP-5c/MP-4a system showed in Figure 2 that although spots # 4 (green), #6 (more intense orange), and #9 (less intense orange) are qualitatively distinguishable by their respective colors, they exhibit a considerable overlapped area (spot #4 with #6 and #6 with #9); therefore, they were considered not well-resolved spots. Nevertheless, after DPPH application, spot #6 appeared very quickly (in the photo taken starting the process (t_0_) a weak yellowish color could be assessed), hinting toward a strong antioxidant activity occurring in such spot. In the case of spot #9, its yellowish color could be assessed only after several hours, which can be interpreted as an antioxidant activity weaker than the one shown by component(s) present in spot #6. Finally, spot #4 never showed antioxidant activity. With such evidence, it could be useful to isolate the global area visualized in yellow in plate with DPPH to obtain a mixture containing components with a noticeable global antioxidant activity. In addition, due to spot #28 remaining without displacement after both chromatographic developments, it was considered a non-well-resolved spot that possibly contained more than one component. Nevertheless, after the application of the DPPH, a very defined pale yellow spot appeared, which demonstrates that, regardless of the number of components, it is a mixture (or a pure component) potentially useful thanks to its antioxidant activity.

## 4. Conclusion

As a first step in the identification of the antioxidant compounds contained in methanolic extracts of* Spirulina platensis*, a simple and fast 2D-TLC biosystem was developed. The proposed experimental system allowed a suitable separation and localization of such type of components, whose dispersion on the plate was favored by the use of NH_4_OH as a phase modifier. The last system was intentionally designed to be scaled to preparative TLC plates, which is a preliminary stage to the identification of components; at this time, we are working on that stage.

## Figures and Tables

**Table 1:** Relative volumetric proportion for the mobile phases used to perform the 1D-TLCs and 2D-TLCs.

**Mobile phase**	**Solvent 1(*δ***_**1**_ **∗** **): Solvent 2 (*δ***_**2**_ **∗** **): modifier**	**Relative volumetric proportion**
**MP-1**	AcO (20.3): n-Hx (14.9): non-used	3:7:0
**MP-2**	AcO (20.3): n-Hx (14.9): non-used	1:1:0
**MP-3**	AcO (20.3): EA (18.6): non-used	1:1:0
**MP-4a**	IOH (23.5): EA (18.6): non-used	0.16:1:0
**MP-4b**	IOH (23.5): EA (18.6): non-used	1:1:0
**MP-4c**	IOH (23.5): EA (18.6): non-used	1:0.16:0
**MP-5a**	IOH (23.5): EA (18.6): NH_4_OH^*∗∗*^	0.16:1:0.25
**MP-5b**	IOH (23.5): EA (18.6): NH_4_OH^*∗∗*^	1:1:0.25
**MP-5c**	IOH (23.5): EA (18.6): NH_4_OH^*∗∗*^	1:0.16:0.25

^*∗*^Solubility parameter (*δ*_i_; expressed in (J/cm^3^)^1/2^) [38]. *∗∗*pK_b_= 4.75.

**Table 2:** Retention factor (R_F_) values of spots observed in the 1D-TLC plates, developed using the indicated mobile phases.

**MP-1**				**MP-2**					**MP-3**		
	**VL**		**UV** **L**_366_		**VL**		**UV** **L**_366_		**VL**		**UV** **L**_366_
1	0.97	12	0.76	1	0.98	7	0.97	1	0.84	8	0.84
2	0.93*∗*	13	0.66	2	0.93	8	0.79*∗*	2	0.82	9	0.71*∗*
3	0.90*∗*	14	0.47	3	0.87*∗*	9	0.70*∗*	3	0.78	10	0.62*∗*
4	0.84*∗*	15	0.41	4	0.76*∗*	10	0.26*∗*	4	0.68*∗*	11	0.36*∗*
5	0.79	16	0.37	5	0.07	11	0.08	5	0.10*∗*	12	0.13*∗*
6	0.69	17	0.07	6	0.00	12	0.03	6	0.04	13	0.04
7	0.58					13	0	7	0.00	14	0
8	0.50										
9	0.11										
10	0.04										
11	0.00										

**MP-4a**				**MP-4b**					**MP-4c**		
	**VL**		**UV** **L**_366_		**VL**		**UV** **L**_366_		**VL**		**UV** **L**_366_

1	0.92	7	0.92	1	0.83	8	0.83	1	0.89	8	0.81*∗*
2	0.84	8	0.79*∗*	2	0.77*∗*	9	0.71*∗*	2	0.87	9	0.59*∗*
3	0.51*∗*	9	0.23*∗*	3	0.69*∗*	10	0.53	3	0.77*∗*	10	0.39*∗*
4	0.43*∗*	10	0.19*∗*	4	0.32	11	0.33	4	0.70*∗*	11	0.18*∗*
5	0.07	11	0.04	5	0.11*∗*	12	0.22*∗*	5	0.49*∗*	12	0.00
6	0.00	12	0.00	6	0.06	13	0.07	6	0.04		
				7	0.00	14	0	7	0		

**MP-5a**				**MP-5b**				**MP-5c**			
	**VL**		**UV** **L**_366_		**VL**		**UV** **L**_366_		**VL**		**UV** **L**_366_

1	0.94	16	0.92	1	0.96	16	0.93	1	0.96	16	0.93
2	0.92	17	0.44*∗*	2	0.93	17	0.70*∗*	2	0.93	17	0.70*∗*
3	0.88	18	0.37*∗*	3	0.91	18	0.46	3	0.91	18	0.59*∗*
4	0.80*∗*	19	0.29*∗*	4	0.58*∗*	19	0.38*∗*	4	0.77*∗*	19	0.49*∗*
5	0.41*∗*	20	0.26*∗*	5	0.54*∗*	20	0.28	5	0.74*∗*	20	0.43*∗*
6	0.36*∗*	21	0.16*∗*	6	0.50*∗*	21	0.16*∗*	6	0.68*∗*	21	0.39*∗*
7	0.32*∗*			7	0.42	22	0.09*∗*	7	0.52*∗*	22	0.32*∗*
8	0.24*∗*			8	0.32*∗*	23	0.00	8	0.50*∗*	23	0.27
9	0.20*∗*			9	0.26			9	0.48*∗*	24	0.17*∗*
10	0.13			10	0.21*∗*			10	0.42*∗*	25	0.04*∗*
11	0.11*∗*			11	0.18*∗*			11	0.38*∗*		
12	0.10*∗*			12	0.14*∗*			12	0.30*∗*		
13	0.09*∗*			13	0.07			13	0.28		
14	0.04			14	0.03			14	0.11*∗*		
15	0.00			15	0.00			15	0.00		

^*∗*^Spots considered as well resolved.

**Table 3:** Retention factor (R_F_) values observed in 2D-TLC plates, developed with the indicated mobile phases.

**VL**			**UVL**_**366**_		
**Spot**	**MP-1**	**MP-1**	**Spot**	**MP-1**	**MP-1**
1	0.74	0.76*∗*	**12**	0.59	0.59*∗*
2	0.66	0.66*∗*	**13**	0.56	0.56*∗*
3	0.62	0.62*∗*	**14**	0.52	0.49
4	0.58	0.53*∗*	**15**	0.41	0.38
5	0.55	0.49	**16**	0.30	0.26
6^+^	0.47	0.44*∗*	**17**	0.15	0.15*∗*
7	0.38	0.37	**18**	0.06	0.12*∗*
8^+^	0.34	0.30*∗*	**19**	0.04	0.02
9	0.31	0.26	**20**	0.00	0.00
10	0.03	0.02			
11	0.00	0.00			

	**MP-5c**	**MP-4a**		**MP-5c**	**Mp-4a**

1	0.79	0.79	**19**	0.46	0.59
2	0.74	0.86	**20**	0.46	0.09*∗*
3	0.68	0.59*∗*	**21**	0.38	0.06*∗*
4	0.64	0.69	**22**	0.34	0.35
5	0.64	0.54*∗*	**23**	0.26	0.35
6	0.60	0.71	**24**	0.26	0.03*∗*
7	0.60	0.53*∗*	**25**	0.16	0.02
8	0.56	0.88*∗*	**26**	0.11	0.00*∗*
9	0.56	0.68	**27**	0.05	0.00*∗*
10	0.50	0.43*∗*	**28**	0.00	0.00
11	0.45	0.48	**2**9^+^	0.20	0.26
12^+^	0.45	0.29*∗*			
13	0.38	0.43*∗*			
14^+^	0.38	0.18*∗*			
15	0.30	0.09*∗*			
16^+^	0.22	0.00*∗*			
17	0.15	0.00			
18	0.04	0.00			

^*∗*^Spots considered as well resolved.

^+^Spots considered as well-resolved and containing compounds with antioxidant activity.

## Data Availability

The data used to support the findings of this study are available from the corresponding author upon request.
